# Connecting Athletes’ Self-Perceptions and Metaperceptions of Competence: a Structural Equation Modeling Approach

**DOI:** 10.1515/hukin-2015-0047

**Published:** 2015-07-10

**Authors:** Jose A. Cecchini, Javier Fernández-Rio, Antonio Méndez-Giménez

**Affiliations:** 1 University of Oviedo; Faculty of Teacher Training and Education; Department of Educational Sciences, Spain.

**Keywords:** perceived competencies, goal orientations, metaperceptions

## Abstract

This study explored the relationships between athletes’ competence self-perceptions and metaperceptions. Two hundred and fifty one student-athletes (14.26 ± 1.89 years), members of twenty different teams (basketball, soccer) completed a questionnaire which included the Perception of Success Questionnaire, the Competence subscale of the Intrinsic Motivation Inventory, and modified versions of both questionnaires to assess athletes’ metaperceptions. Structural equation modelling analysis revealed that athletes’ task and ego metaperceptions positively predicted task and ego self-perceptions, respectively. Competence metaperceptions were strong predictors of competence self-perceptions, confirming the atypical metaperception formation in outcome-dependent contexts such as sport. Task and ego metaperceptions positively predicted athletes’ competence metaperceptions. How coaches value their athletes’ competence is more influential on what the athletes think of themselves than their own self-perceptions. Athletes’ ego and task metaperceptions influenced their competence metaperceptions (how coaches rate their competence). Therefore, athletes build their competence metaperceptions using all information available from their coaches. Finally, only task-self perfections positively predicted athletes’ competence self-perceptions.

## Introduction

The social cognitive theory (Bandura, 1986) argues that individuals both influence and are influenced by different personal, environmental, and behavioural factors. Researchers have stressed the need to distinguish between direct perceptions or first-order expectations, and metaperceptions or second-order expectations (Troyer and Younts, 1997). Direct perceptions relate to the beliefs that individuals hold for themselves or others, while metaperceptions are estimations formed by people regarding the thoughts of others (Kenny and Acitelli, 2001). In other words, metaperceptions are perceptions of how other people view us (Kaplan et al., 2009) and their role has long been the subject of research within social psychology (Kenny and DePaulo, 1993). In sport contexts, Adie and Jowett (2010, p. 2754) highlighted the importance of metaperceptions in shaping the quality of coach athlete interactions: “the coach’s and the athlete’s interrelated cognitions, emotions, and behaviours are captured through the interpersonal constructs of commitment, closeness, and complementarity”. However, athletes’ metaperceptions have not been widely researched.

Sport can be considered an activity where power asymmetry exists since “outcomes are dependent upon someone in a more powerful position” (Kaplan et al., 2009). According to this idea, athletes could be considered outcome-dependent individuals. Coaches and athletes do not interact at the same level, since the first ones have a strong influence over the second’s sport career. Playing opportunities, performance-related feedback or rewards/punishments are important elements in an athlete’s global development in sport, and they strongly depend on his/her coach’s actions. In this type of context, there seems to be a hierarchical nature of metaperception formation since individuals are more concerned about the evaluations of those more powerful, knowledgeable or expert (Kaplan et al., 2009). Traditional views on metaperception formation consider that individuals rely on their own default self-perceptions. They look inward, not outward, and infer that their interaction partners view them as they view themselves (Kenny and DePaulo, 1993). However, outcome-dependent contexts, such as sport, are more likely to promote greater motivation to know how others view us (Kaplan et al., 2009), but subordinates are more responsive to leaders’ views than the opposite (Snodgrass, 1992). In these types of settings, Kaplan et al. (2009) believe that there is an atypical metaperception formation since it derives not from default self-perceptions, but from influential others’ perceptions. They consider that metaperceptions influence self-perception when individuals are dependent on their powerful interaction partners (Kenny and DePaulo, 1993). In order to reduce uncertainty and increase predictability, individuals try to evaluate others’ thoughts and behaviours to adjust to their expectations (Hall et al., 2006). They tend to adopt a “bottom-up processing strategy” (Kaplan et al., 2009), to obtain more information from their more important counterparts to better understand their behaviours. However, these ideas have not been researched in outcome-dependent settings such as sport.

Motivation has been widely researched as one of the key elements that can influence outcomes in sport. The achievement goal theory (AGT; Nicholls, 1984) has been fundamental to be able to understand the meaning that athletes attach to achievement (success or failure). Basically, achievement goals are divided in two major groups: task and ego (Nicholls, 1984). Task-oriented individuals desire to improve their skills and focus on the development of competence comparing their performance with their own previous performances (their perception of competence is self-referenced). Ego-oriented individuals desire to be better than others and focus on the demonstration of superior competence comparing their performance with other individuals’ performance (their perception of competence is normative). According to Duda and Nicholls (1992, p. 291) “one might expect a moderate association between perceived ability and ego orientation” in sport settings.

Another major framework used to study motivation in achievement contexts such as sport is Harter’s competence motivation theory (1981). It proposes that youngsters become motivated to engage in sport to demonstrate competence. Therefore, individuals’ perceptions of competence seem to be fundamental to enjoy involvement in sport activities, and those with positive perceptions tend to show higher motivation levels (Ryan and Deci, 2000). Harter (1978) included coaches as those significant ones (besides parents, friends or teammates) who are influential on athletes’ perceptions of competence. Horn (1985) found that coaching behaviours were stronger mediators than skill improvement on athletes’ self-perceptions. Black and Weiss (1992) found similar results in a group of young swimmers. Coaches’ praise, information and encouragement influenced their athletes’ perceived competence. Besides coaches, athletes’ own perceptions of competence are influenced by their ability level (Harter, 1981). In a group of female athletes Horn (1985) found that ability significantly influenced perceived competence. Similarly, Allen and Howe (1998) found that higher ability, as well as praise and information from coaches, were linked to greater satisfaction in a group of female adolescent athletes. Research tells us that athletes’ perceptions of competence will strongly affect their engagement in sport, their goals and behaviours (i.e., time spend in an activity), effort exerted, and persistence (Duda and Hall, 2001). Therefore, understanding how individuals build their self-competence perceptions seems fundamental to achieve positive results in outcome-based settings such as sport.

On the other hand, coaches could be considered responsible for the motivational climate developed in sport contexts (other important actors involved are peers, parents or spectators). Research has distinguished two main achievement environments: mastery or task-involving and performance or ego-involving (Ames, 1984). In mastery climates, success and failure are defined in terms of skill mastery and individual improvement. They are task-involving settings that emphasize the process of skill development, effort, and personal improvement. They promote mastery-oriented individuals that try hard even when facing difficulties, show intrinsic interest in the different tasks, and persist over time (Roberts, Treasure, and Balagué, 1998). In performance climates, success and failure are defined in normative terms with an emphasis on outperforming teammates and opponents. They are ego-involving environments that focus on the outcomes and doing better than others. They promote performance-oriented individuals that are worried about being judged as better than their partners. Coaches’ greater emphasis on mastery climates has been related to higher athletes’ ability perceptions (Weiss et al., 2009).

**Figure 1:**
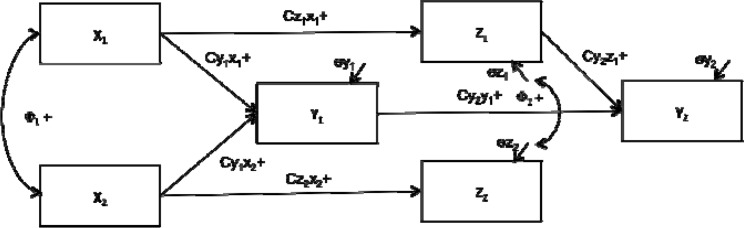
Hypothesized model containing the parameters of the Path analysis. x_1_ = Task Metaperception; x_2_ = Ego Metaperception; z_1_ = Task Self-perception; z_2_ = Ego Self-perception; y_1_ = Competence Metaperception; y_2_ = Competence Self-perception.

Previous research has explored the atypical metaperceptive formation in outcome-dependent educational contexts (Kaplan et al., 2009). However, no studies have addressed this issue in sport settings, where coaches are in a powerful position. Moreover, there has been a call to study the coach-athlete relationship to understand motivation in sport (Adie and Jowett, 2010). Based on the aforementioned, the primary goal of this study was to explore, through a path analysis, the relationships between athletes’ competence self-perception and metaperception. The first hypothesis was that task metaperceptions will positively influence task self-perceptions (Cz1x1 +), while ego metaperceptions will positively influence ego self-perceptions (Cz2x2 +). The second hypothesis was that athletes’ competence metaperceptions will be linked to task (Cy1x1 +) and ego metaperceptions (Cy1x2 +). Finally, the third hypothesis was that athletes’ competence self-perceptions will be dependent upon task self-perceptions (Cy2z1 +) but, more important, upon competence metaperceptions (Cy2y1 +). We believed that how others view us is more influential on how we see ourselves than our self-perceptions (Figure 1).

## Material and Methods

### Participants

Two hundred and fifty one student-athletes (116 women, 135 men, *age* = 14.26 ± 1.89, age range: 11–17 years) agreed to participate in the present study. They were members of a total of 20 different teams (10 basketball, 10 soccer) located in the northern part of Spain.

### Measures

#### Self-perception of success

The Perception of Success Questionnaire (POSQ; Roberts et al., 1998) was used to measure each participant’s task or ego orientation. It is a 12-item assessment instrument grouped in two subscales (six items each). Each item is headed by the stem: “When playing my sport, I feel most successful when...”. Items in the task subscale include: ”I perform to the best of my ability”, while items on the ego subscale include: “I outperform my opponents”. Cervelló et al. (1999) assessed the validity of this instrument for Spanish contexts. The task self-perception (*α* = .75) and ego self-perception (*α* = .85) scales were internally consistent in this study.

#### Metaperception of success

In order to assess athletes’ success metaperceptions, the Perception of Success Questionnaire (POSQ; Roberts et al., 1998) was used again. The stem of each item was changed to: “When playing my sport, my coach feels that I am successful when...”. This instrument had been validated for Spanish contexts by Cecchini et al. (2014). The internal reliability of this instrument was found to be acceptable in this study (*α* = .84 for task meta-perception, and *α* = .87 for ego meta-perception scales, respectively).

#### Competence self-perception

The 5-item Competence subscale of the Intrinsic Motivation Inventory (IMI; McAuley et al., 1989) was used to assess athletes’ competence self-perceptions. The generic label “activity” was reworded to reflect the nature of the current activity: basketball and soccer. Participants were asked to rate their agreement/disagreement with several statements (e.g. “I am pretty skilled at basketball”). Balaguer et al. (2008) proved the validity of this instrument for Spanish contexts. The Cronbach’s alpha coefficient was acceptable in this study (*α* = .85).

#### Competence metaperception

In order to assess athletes’ competence metaperceptions, the competence subscale of the Intrinsic Motivation Inventory (IMI; McAuley et al., 1989) was used again. The stem “My coach believes that…” was added to the subscale. This instrument had been validated for Spanish contexts by Cecchini et al. (2014). Its internal reliability in this study was found adequate (*α* = .83).

### Procedures

The implementation of the research project involved three steps: first, permission from the Ethics Committee of the researchers’ University and the student-athletes’ club was obtained. Second, all the participants’ parents signed an informed consent form (all of them were under 18 years of age). Third, a specifically designed questionnaire, which included all the subscales described earlier, was administered by one of the researchers prior to regularly scheduled training sessions. Participants were asked to respond to all questions on a 5-point Likert scale ranging from 1 (strongly disagree) to 5 (strongly agree). Prior to questionnaire administration, athletes were told that their responses would be kept confidential. They were also informed that their coaches would not have access to their answers. Researchers encouraged students to answer truthfully, and informed them that they could withdraw from the process at any time.

#### 

##### Statistical Analysis

Analyses were conducted using the SPSS 18.0 and the EQS 6.2 programs. Before proceeding with hypothesis testing, the statistical assumptions were tested (i.e. normality, linearity, and multicollinearity). In addition, descriptive statistics and bivariate correlations were conducted to explore the trends and relationships among variables.

The hypothesized model was tested through a Path analysis (EQS 6.2). This is the most widely used technique to test the relationship among variables. Given that preanalyses revealed substantial multivariate kurtosis (4.84), analysis were based on the Satorra-Bentler scaled chi-square statistic (S-B χ^2^; Satorra and Bentler, 1994), since it serves as a correction for χ^2^ when distributional assumptions are violated. Previous research had shown that kurtosis severely affects tests of variance and covariance (DeCarlo, 1997).

In testing the initial model, evaluation of goodness-of-fit to the sample data was determined on the basis of multiple criteria (Byrne, 2008): the Comparative Fit Index (*CFI), the Root Mean-Square Error of Approximation (*RMSEA), and the Standardized Root Mean Square Residual (SRMR). The *CFI represents the robust version of the CFI in that its computation is based on the S-Bχ^2^ statistic. It ranges in value from 0 to 1.00. The *RMSEA is a robust version of the RMSEA, and it takes into account the error of approximation in the population. Values less than .05 indicate good fit, and values as high as .08 represent reasonable errors of approximation in the population. To complete the analysis, the 90% confidence interval provided for *RMSEA was considered. Lastly, the SRMR is the average standardized residual value derived from fitting the hypothesized variance covariance matrix to that of the sample data. Its value ranges from 0 to 1.00, with a value less than .08 being indicative of a well-fitting model.

To examine which parameters of the hypothesized model were invariant across the two samples (basketball and soccer), a multistep analysis of invariance was employed. According to Byrne (1998), the first step involves establishing an appropriate baseline model, which is tested across the samples. This is a non-invariant step, and it provides a critical base for subsequent model comparisons. Next, structural weights are constrained to be invariant across groups. The subsequent step involves constraining the covariance matrix to equivalence across groups, with the structural weight still constrained. Finally, the uniqueness (error) is set to equivalence across groups, with the structural weight and covariances still constrained.

## Results

### Descriptive Statistics and Bivariate Correlations

Table 1 shows Cronbach’s alpha coefficients of the different subscales, means, and standard deviations, as well as bivariate correlations among all variables. Cronbach’s alphas were above .70 in all subscales, which indicated that the internal consistency of all of them was acceptable in this study (Nunnally and Bernstein, 1994). The highest mean scores were obtained in task metaperceptions and the lowest in ego self-perceptions. Correlation analysis showed that all variables were positively correlated. As expected, the highest correlation scores were measured between coaches and athletes’ task self-perceptions and metaperceptions and athletes’ ego self-perceptions and metaperceptions.

### PATH Analysis

**Figure 2:**
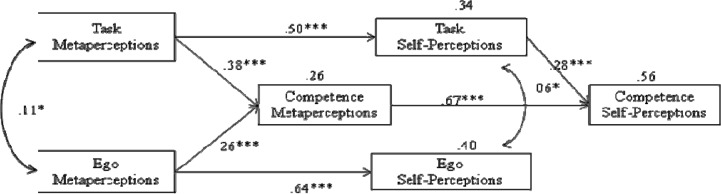
Tested model depicting the predicted relationships among variables. *p< .05; ***p< .001

The initial testing of the hypothesized model yielded a good fit to the data: S-Bχ^2^ (7)= 9.44. p = .222; χ^2^; *CFI= 1.00; SRMR= .03; *RMSEA= .037; 90% CI= .037 (.000–.092). Figure 2 shows the tested model with the predicted relationships among variables. Task and ego metaperceptions positively influenced competence metaperceptions. Task and ego metaperceptions positively influenced task and ego self-perceptions, respectively. Finally, the direct effect of competence metaperceptions was a stronger predictor of competence self-perceptions when compared to task self-perceptions.

### Multistep Analysis of Invariance

Results presented in Table 2 show that the tested models had acceptable fit indexes. Besides Δχ^2^, Δ*CFI was also used. According to Cheung and Rensvold (2002), when Δ*CFI is equal to or lower than −.01, the invariance null hypothesis cannot be rejected. Therefore, these results reinforce the hypothesized model.

## Discussion

The purpose of this study was to explore a motivational sequence hypothesizing relationships between athletes’ competence self-perceptions and metaperceptions (coaches’ views on their competence) testing the atypical metaperception formation in sport settings. The results of the study provided strong support for our hypotheses and the atypical metaperceptive processing. Athletes’ competence metaperceptions were the strongest predictors of their competence self-perceptions. Task and ego metaperceptions positively predicted athletes’ competence metaperceptions, while task meta and self-perceptions and ego meta and self-perceptions were also linked, respectively. Finally, we also found that both task and ego metaperceptions influenced competence metaperceptions (how coaches view them), while only task self-perceptions were linked to competence self-perceptions.

Our first hypothesis was that task metaperceptions would positively influence task self-perceptions, while ego metaperceptions would positively influence ego self-perceptions. Our results support this idea. This finding is in line with the atypical metaperception formation theory (Kaplan et al., 2009) which considers that metaperceptions drive self-perceptions in outcome-dependent contexts, and sport is one of them. Individuals try to decode the significance of the behaviours displayed by the more powerful persons in the context, which is usually the coach in sport settings, to form their self-perceptions. Previous research has linked contextual and situational variables within the AGT (Cecchini et al., 2005), which reinforces our results. Certainly, athletes’ views on how their coaches see them can be very influential on how they see themselves. Coaches’ comments, behaviours, decisions or feedback are scrutinized by their athletes trying to understand what their thoughts are on them, because coaches play an important role in the athlete’s sport career.

Our third hypothesis was directly linked to the first one. We hypothesized that athletes’ competence self-perceptions would be dependent upon their task self-perceptions, but more important, upon their competence metaperceptions. Results showed that competence metaperceptions were a stronger predictor than task self-perceptions on the athletes’ competence self-perceptions. Participants believed that how others view them (coaches) was more influential on how they saw themselves than their own self-perceptions. Once again, this finding supports the atypical metaperception formation theory in sport, an outcome-dependent setting where individuals do not rely on default self-perceptions, but engage in a bottom-up strategy to evaluate their coaches’ behaviours (Kaplan et al., 2009). In these contexts, in order to reduce uncertainty and increase predictability, athletes try to evaluate their coaches’ thoughts and behaviours to adjust to their expectations (Hall et al., 2006). Therefore, their perceived competence self-perception relies heavily on what they think coaches think of them (metaperception) more than what they think of themselves. Situations such as those of the sporting field, where an individual’s outcomes depend on what significant others (e.g., coaches) think of him/her, seem to promote concern about how those others see him/her (Hinde, 1995). According to Felson (1993), athletes’ self-perceptions of competence are heavily influenced by how others view and evaluate them, and coaches seem to play an important role, since they are likely to communicate their feelings about the athletes’ strengths and weaknesses, and behave accordingly.

The role of significant others in sport (e.g., teammates, coaches or parents) has been highlighted as very influential in previous research (Amorose, 2003). It has been suggested that individuals tend to see themselves as they believe others see them, and our results support this idea. To gather complete understanding of relational processes, researchers should account for “one’s own expectations regarding self and other and one’s beliefs about the expectations other holds for self and other” (Troyer and Younts, 1997, p. 696), and coaches are significant others in the world of sport. Moreover, they are powerful individuals in their athletes’ sporting lives, and their behaviours are carefully considered by athletes. This equation leads to power asymmetry. When power asymmetry exists, individuals closely dissect the important person’s motives and behaviours (Stevens and Fiske, 2000). Perceived competence can play a main role in predicting motivation and behaviour in sport and exercise (Roberts et al., 2007), and our results show that coaches’ influence on students’ self-perceptions of competence is significant. Therefore, it seems crucial to examine the coach– athlete relationship, their interrelated cognitions, emotions or behaviours (Adie and Jowett, 2010), and our results show that athletes’ competence metaperceptions (how coaches view them) supply valuable information regarding athletes’ competence self-perception formation.

Our second hypothesis was that task and ego metaperceptions would positively predict athletes’ competence metaperceptions. Previous research has showed that greater emphasis placed by coaches on a mastery climate is related to higher athletes’ ability perceptions (Weiss et al., 2009). However, our results indicated that not only task, but also perceptions of a coaching ego climate positively influenced, although moderately, athletes’ competence metaperceptions. These results can be explained by the asymmetrical metaperception formation theory (Kaplan et al., 2009). Participants decided if they were competent or incompetent based on both, task and ego metaperceptions. Ego metaperceptions are normative and they seemed to influence how individuals believed significant others (e.g. their coaches) valued their competence. Certainly, athletes tend to integrate all types of comments and behaviours of their coaches, no matter if they are task oriented (self-referenced) or ego oriented (normative). Athletes use both types of inputs to build their competence metaperceptions (how they think their coaches view them) to adjust and build their competence self-perceptions.

Our results also showed that only task self-perceptions were linked to athletes’ competence self-perceptions. This finding indicates that participants were task-oriented individuals. They were athletes with the desire to improve their skills focusing on the development of competence, and comparing their performance with their own previous performance (Ryan and Deci, 2000). The tested model showed that this orientation positively influenced the athletes’ competence self-perceptions, along with their competence metaperceptions, and it confirmed the moderate link between perceived ability and ego orientation (Duda and Nicholls, 1992).

Considering these last two findings, we would like to highlight that in this group of athletes, ego metaperceptions significantly influenced their competence metaperceptions (although moderately), while ego self-perceptions did not influence their competence self-perceptions.

Our results support the idea that outcome-dependent contexts such as sport, where power asymmetry exists, promote an atypical metaperception formation of competence, since it derives, mainly, not from default self-perceptions, but from influential partners like coaches. That is: metaperceptions drive self-perceptions. Both task and ego metaperceptions influenced competence metaperceptions (how coaches view them), while only task self-perceptions were linked to competence self-perceptions. Coaches should be aware of their influence over their athletes.

Despite the positive results obtained, this study also holds some limitations. The cross-sectional nature of the study does not allow us to discuss possible causal links between the observed variables. The homogeneity of the sample is another limitation. Participants were young student-athletes training and competing in the same area. Therefore, future studies should test our findings with different age groups. Older and/or younger athletes could draw a different picture of self-perception formation. The skill level of the athletes could be another issue to consider: professionals or high-level athletes could generate different results. The type of sport should also be taken into account. Participants in our study were team sport athletes, and our findings should be tested in individual sport athletes. Finally, other variables such as perceived motivational climate generated by the coach should be examined to obtain a global model of athletes’ competence metaperceptions.

This is the first study to assess the atypical metaperception formation in sport, where the metaperception drives self-perfection. Previous research had tested this theory in educational contexts, but our study broadens and deepens its scope. The key message of this study is that sport contexts demand greater attention toward significant others’ presumed interpretations and reactions. How coaches value their athletes’ competence is more influential on what the athletes think of themselves than their own self-perceptions. They do not seem to rely on default self-perceptions, but engage in bottom-up strategies to evaluate their coaches’ behaviours. Therefore, in outcome-dependent contexts such as sport where power asymmetry is very strong, athletes’ competence self-perceptions are strongly influenced by their competence metaperceptions, in particular those coming from their coaches. Athletes’ competence metaperceptions drive their competence self-perceptions. A second remarkable finding was that athletes’ ego and task metaperceptions influenced their competence metaperceptions (how coaches rate their competence). These athletes seemed to build their competence metaperceptions using all information available from their coaches, no matter if it was task or ego-oriented. The idea is that any type of environment created by the coaches (task or ego-involving) will impact the athletes’ competence metaperceptions and accordingly, their competence self-perceptions. A final significant finding was that only task self-perceptions were linked to competence self-perceptions. When these athletes construct their competence self-perceptions, they were significantly influenced only by their task orientations.

## Figures and Tables

**Table 1:** Cronbach’s alphas, means, standard deviations, and bivariate correlations in all variables

	*α*	M	SD	1	2	3	4	5
1. Task Metaperceptions	.84	4.19	.72					
2. Ego Metaperceptions	.87	2.91	.94	.16^*^				
3. Task Self-perceptions	.75	4.09	.62	.60^**^	.14^*^			
4. Ego Self-perceptions	.85	2.82	.95	.19^**^	.64^**^	.25^**^		
5. Competence Metaperceptions	.83	3.56	.77	.41^**^	.38^**^	.28^**^	.32^**^	
6. Competence Self-perceptions	.85	3.71	.79	.44^**^	.22^**^	.41^**^	.24^**^	.72^**^

* p < .01;

** p <.05

**Table 2:** M_1_ = Model 1: without restrictions; M_2_ = Model 2: structural weight invariance; M_3_ = Model 3: structural covariance invariant; M_4_ = Model 4: structural residual invariance

Model	S-Bχ^2^	df	Δχ^2^	Δdf	*CFI	SRMR	*RMSEA (90% CI)
M_1_	23.29	14	-	-	.98	.04	.052 (.000 –.087)
M_2_	28.42	20	5.13	6	.98	.04	.041 (.000 –.073)
M_3_	32.72	23	4.3	3	.98	.06	.041 (.000 –.071)
M_4_	45.16	28	12.44	5	.97	.07	.050 (.019 –.075)

S-Bχ^2^: Satorra-Bentler Scaled Chi-Square; df: Degrees of Freedom;

Δχ^2^: Standardized Chi-Aquare; Δdf: Standardized Degrees of Freedom;

*CFI: Comparative Fit Index; SRMR: Standardized Root Mean Square Residual;

*RMSEA: Root Mean-Square Error of Approximation; CI: Confidence Interval.
